# Semaglutide-Associated Ischemic Colitis in a Patient Without Traditional Risk Factors: A Case Report

**DOI:** 10.7759/cureus.78832

**Published:** 2025-02-10

**Authors:** Sommer Sekhon, John Bacon, Ibaadat Sukhmanii Kahlon

**Affiliations:** 1 Biology, The Smittcamp Family Honors College, California State University (CSU), Fresno, USA; 2 Internal Medicine, Saint Agnes Medical Center, Fresno, USA; 3 Internal Medicine, University of Medicine and Health Sciences, Basseterre, KNA

**Keywords:** drug induced colitis, drug-induced colitis, glp1-ra, ischemic colitis, ozempic, semaglutide

## Abstract

This report describes the case of a 43-year-old female patient presenting with left lower quadrant pain and bloody diarrhea, initially managed as infectious colitis. Despite treatment, symptoms persisted, leading to repeat imaging showing mild pancolitis with colon mucosal hyperenhancement and wall thickening. Admission for intravenous hydration and subsequent colonoscopy revealed findings consistent with ischemic colitis. Semaglutide (Ozempic), which the patient had been taking, was discontinued, and symptoms resolved post discharge without semaglutide use for several months. This suggests a potential association between semaglutide and ischemic colitis in a patient without known risk factors. This case underscores the importance of considering medication-induced colitis in patients presenting with persistent gastrointestinal symptoms, particularly in the absence of typical risk factors.

## Introduction

Ischemic colitis is a condition characterized by reduced blood flow to the colon, leading to inflammation and injury of the intestinal wall [1]. Age > 60 years, atherosclerosis, smoking, chronic kidney disease (CKD), and atrial fibrillation are well-established factors associated with an increased risk of developing ischemic colitis. Ischemic colitis in younger individuals without these traditional risk factors is rare and often presents a diagnostic challenge [2].

Semaglutide (Ozempic), a glucagon-like peptide-1 receptor agonist used primarily for the management of type 2 diabetes, has been associated with gastrointestinal side effects, but its potential role in inducing ischemic colitis is not well documented. In this report, we describe the case of a 43-year-old woman who developed ischemic colitis potentially associated with semaglutide use, highlighting the need for clinicians to consider medication-induced ischemic colitis even in patients without conventional risk factors.

## Case presentation

**Figure 1:**
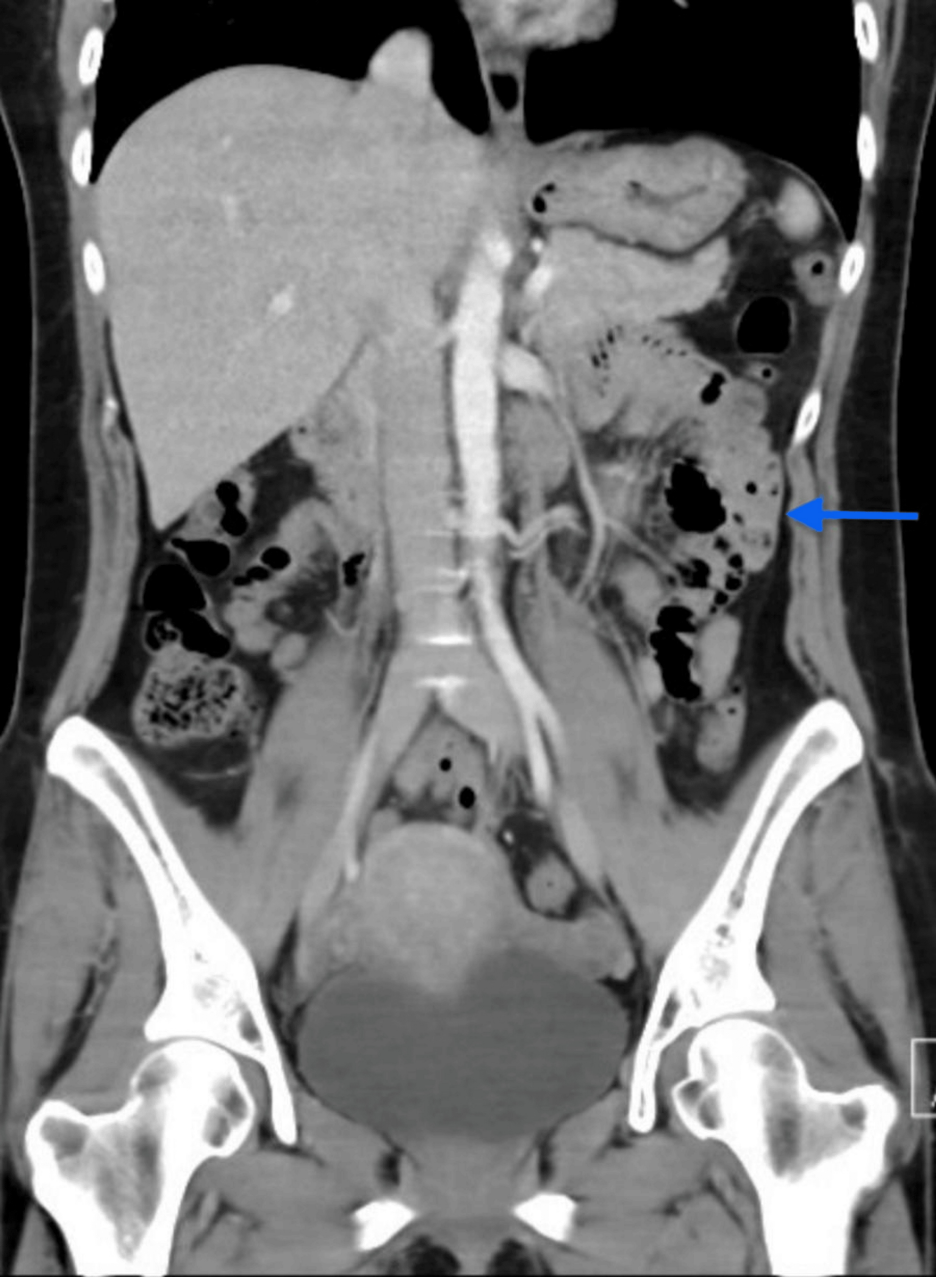
CT abdomen pelvis demonstrating pancolitis Bowel wall thickening and mucosal enhancement demonstrated by the blue arrow

A 43-year-old female patient presented with left lower quadrant pain, and bloody diarrhea. She had a past medical history of hypothyroidism. She initially had one day of lower abdominal pain and bloody diarrhea for which she had visited the emergency department. She had a normal white blood cell count, hemoglobin was 11.8 g/dL (12.1-15.1 g/dL), and transaminases were within normal limits. The initial CT scan in the emergency department did not show any intestinal abnormalities, including colitis. She was discharged with 10 days of metronidazole and ciprofloxacin. However, she only took five days of antibiotics and after this, she experienced no more symptoms for 10 days. However, her symptoms returned, and she presented to the emergency department again. The CT abdomen done this time showed pancolitis with mucosal hyperenhancement and wall thickening involving the entire colon (Figure 1).

The patient's stool initially tested positive for *Campylobacter*. After three days of hydration, azithromycin treatment, and electrolyte replenishment, the stool tested negative for *Campylobacter*. She was discharged on ferrous sulfate for 15 days, and her semaglutide was discontinued. 

She followed up with her gastroenterologist after discharge. Her symptoms had not recurred. Colonoscopy was performed after four months to rule out inflammatory bowel disease or other pathologies. The exam showed a grossly normal colon. Biopsy showed a mild intraepithelial lymphocytosis, which was nonspecific and could be consistent with ischemic colitis, lymphocytic colitis, and medication-induced changes. Following this, she was restarted on her semaglutide.

**Figure 2:**
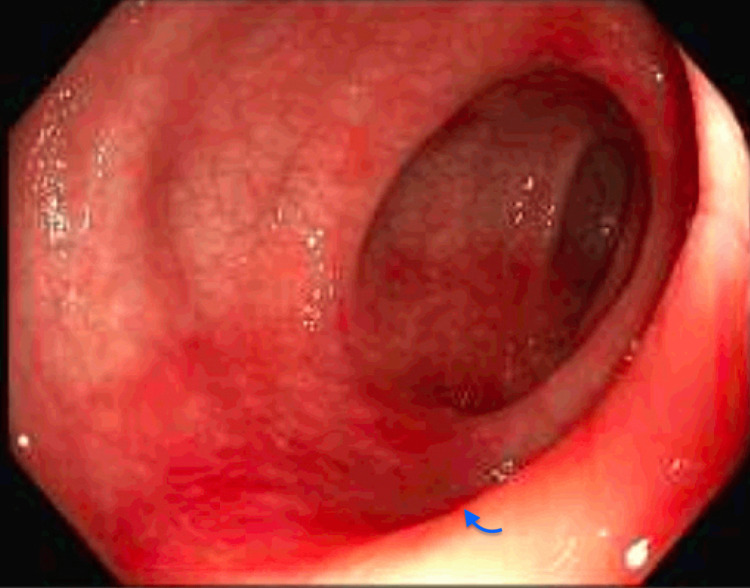
Diffuse mild inflammation seen on colonoscopy Blue arrow demonstrating areas of inflammation

After six months, she returned to the hospital with symptoms of lower abdominal cramping, bloody diarrhea, and vomiting. Repeat CT abdomen pelvis did not show any acute abdominal or pelvic pathology. A repeat colonoscopy was performed, and gross examination showed diffuse mild inflammation found from 20-40 cm proximal to the anus secondary to colitis (Figure 2).

**Figure 3:**
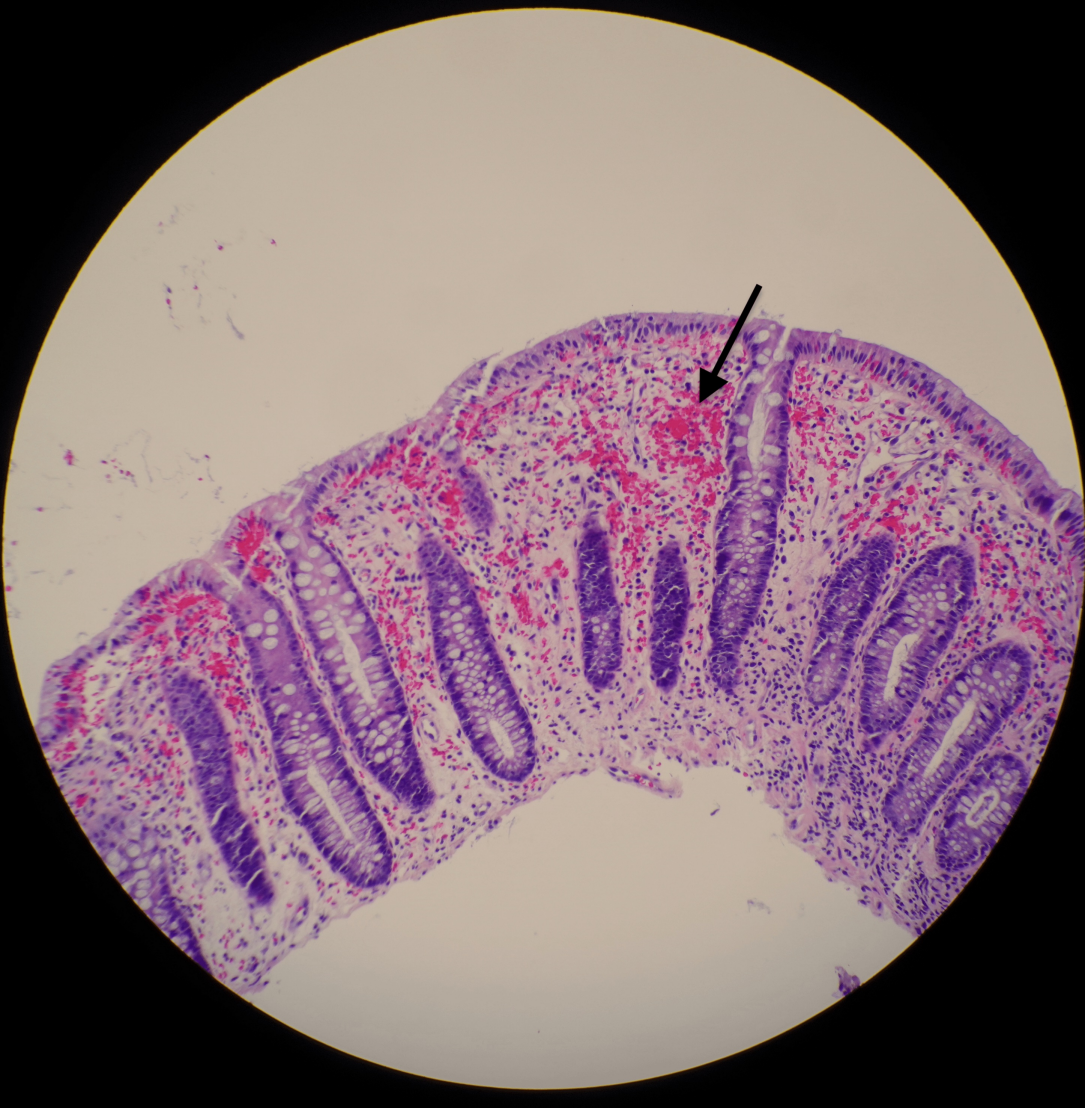
The presence of mucosal necrosis, hemorrhage, and inflammatory cell infiltration suggests ischemic colitis-like changes. Black arrow representing areas of congestion within the mucosa

Biopsies were taken from the terminal ilium cecum, transverse colon, sigmoid colon, and rectum. The sigmoid sample showed mild active colitis, with associated lamina propria fibrosis and extravasated red blood cells, suggestive of ischemic colitis (Figure 3).

The rectal biopsy showed mostly unremarkable large intestinal mucosa with focal epithelial erosion and associated minimal active inflammation. The other samples had no significant pathologic abnormality. Following this, semaglutide was stopped and her symptoms did not return.

## Discussion

The clinical diagnosis of ischemic colitis is made by combining symptoms, signs, radiographic findings, and colonoscopy findings. In this instance, the patient had a sudden onset of abdominal pain, diarrhea, and blood in stools, with characteristic macroscopic and microscopic findings on colonoscopy consistent with the diagnosis [3]. She had an initial episode without the colonoscopy findings, but with *Campylobacter* present in her stool. However, the second episode with these symptoms did not show *Campylobacter *presence and had characteristic findings on colonoscopy. *Campylobacter* stool tests have a small false positive rate. It is possible that the first incidence of symptoms was *Campylobacter*, but in light of a second occurrence with a microscopic appearance consistent with ischemic colitis, it is most likely that both appearances were due to ischemic colitis. The first occurrence of symptoms occurred with nonspecific tissue findings and symptoms and could be consistent with either ischemic colitis or *Campylobacter*; however, the most likely unifying diagnosis for the second occurrence of symptoms was ischemic colitis. These findings are unusual in a patient in her 40s, but could be due to hypovolemia from semaglutide. 

Ischemic colitis is generally considered a disease of geriatric patients and is associated with CKD, atherosclerosis, diabetes mellitus, coagulopathies, vasculitis, and other comorbid conditions. Guidelines from the American College of Gastroenterology state that ischemic colitis increases in likelihood after 49 years of age, but can occur in patients of all ages [3]. Younger patients with ischemic colitis generally have it due to an acute cause such as hypovolemic shock or drug use. In one study examining ischemic colitis in adults under 50 years of age, 49% of the patients had no identifiable predisposing factors, but associations between ischemic colitis and both hypovolemia and vasoactive drugs were identified [4]. Semaglutide is a vasoactive drug, and its gastrointestinal side effects may contribute to hypovolemia. 

The most prevalent risk factor for colon ischemia in one study was the use of drugs with a side effect of constipation [5]. Semaglutide frequently has a side effect of constipation, with a 24.2% incidence of this symptom in a secondary analysis of the Semaglutide Treatment Effect in People with obesity (STEP) 1-3 trials [6]. Semaglutide is associated with lower oral intake, which appears to be at least in part due to an increase in satiety and its accompanying gastrointestinal side effects including nausea and vomiting. This reduced intake may lead to dehydration, hypovolemia, and hypotension, which could lead to ischemic colitis. There have been previously described cases of ischemic colitis, even in previously healthy young adults [7]. Another possible indirect mechanism of ischemia may be due to a reduction in the mechanical and neural stimuli that are required for adequate blood flow. Semaglutide is known to delay gastric emptying, which may lead to a reduction in stimuli for further digestion and affect neural control of the intestinal vasculature. However, little literature exists on this and it could be a direction for future research. 

Semaglutide may directly affect vascular tone itself; however, the existing research is conflicting. Glucagon-like peptide-1 (GLP-1) is known to have complex physiological effects on vascular tone, gastrointestinal motility, and central satiety perception. However, there is not much evidence regarding its effects on blood pressure, and the acute administration of GLP-1 in patients with type 2 diabetes did not change systolic or diastolic blood pressure in the radial artery or renal vein compared to controls [8]. In a similar experiment, exogenous GLP-1 administered to patients with type 2 diabetes and individuals with normal glucose tolerance resulted in attenuation in the typical postprandial fall in blood pressure that occurs in these patient populations.

Semaglutide use has increased in popularity over the last several years due to its utility for weight loss, and its antihyperglycemic effects. Thus, more side effects are likely to appear in post-marketing surveillance. At least one other instance of ischemic colitis associated with semaglutide use has been described [9], and more may come to light as the population exposed to this drug increases in size. There are opportunities for further research for understanding the vasoactive properties and physiology of semaglutide use, and how these relate to patient outcomes.

## Conclusions

This case report highlights a rare instance of ischemic colitis potentially linked to semaglutide use in a patient lacking typical risk factors. The patient's symptoms persisted despite initial treatment for infectious colitis, leading to a revised diagnosis based on imaging and colonoscopy findings. Discontinuation of semaglutide resulted in the resolution of symptoms, suggesting a possible association between the medication and ischemic colitis. Clinicians should be vigilant for medication-induced gastrointestinal complications, particularly in patients presenting with atypical symptoms or lacking conventional risk factors for ischemic colitis. Further research and awareness are warranted to better understand the risk profile and management of medication-related gastrointestinal adverse effects in diverse patient populations.
